# Medicine

**Published:** 1893-12

**Authors:** J. E. Shaw


### Progress of the Medical Sciences.

### MEDICINE.

When, in 1889, Brown-Séquard brought before the Society of Biology in Paris his experiences in the use of an organic extract upon his own person, giving rise thereby to the expression of considerable ridicule, he did but record the practice of a principle which he had enunciated in his course at the Faculty of Medicine twenty years before, when he said: "All glands, whether or not provided with excretory ducts, supply to the blood useful substances, the absence of which is manifested after these glands have been extirpated or destroyed by disease."[1] That is to say, that all glands have the common function of forming an internal secretion, which in glands provided with ducts is entirely different from, and independent of, the external secretion discharged from the gland through its duct. The best known example of two such independent secretions are those of the pancreas. Minkowski's researches, especially his latest,[2] prove conclusively that this organ is constantly throwing into the blood some substance (as yet unknown) which prevents the appearance of glucose in the urine, entirely irrespective of the fact whether the external secretion is allowed to pursue its normal course, or is diverted straight from the gland out of the body through an artificial fistula. Furthermore, Hédon's experiment,[3] to the effect that the pancreas of another animal grafted subcutaneously into the original animal, in whom the pancreas had been destroyed, would prevent glycosuria, gave, if possible, an additional proof to the existence of an internal secretion being formed by this gland.

There are also many general and particular reasons for believing that an analogous function is discharged by tissues non-glandular in their structure, so that, as Treviranus said, every structure, so far as its nutrition is concerned, is relatively to the rest of the body in the condition of an excreted substance, or, as Brown-Séquard has more definitely put it, every act of nutrition is always the correlative of an internal secretion. Such a doctrine opens up a vast field for therapeutic experiment in the employment of the extracts obtained from various tissues and organs, but as yet only a limited number of these organic extracts have been used in practice. It will be sufficient for our

[1] *Arch. de Physiol.*, July, 1891.

[2] *Arch. f. exp. Path. u. Pharm.*, vol. xxxi., pp. 85–189, and *Med. Chron.*, Aug., 1893.

[3] *Arch. de Physiol.*, Oct., 1892.

purpose, therefore, to mention those which so far have been most employed; viz., those from the testicle, thyroid, pancreas, kidney, supra-renal capsule, and nerve-substance; those from the lymphatic glands, spleen, bone-marrow, and muscles are as yet less known and tested.

The modes of preparation of these liquid extracts will be found described by Brown-Séquard,[1] or, in more detail, by Dr. C. Éloy[2] and Dr. H. Melville.[3] These liquids can all be administered hypodermatically or in larger doses by the rectum, but only the preparations obtained from the thyroid, thymus, and bone-marrow can be given with effect by the mouth, although in England pancreas is administered by that method also.

1st. **Testicle.**—The mental and physical characteristics which are dependent upon the development and presence of the testicles and their secretions are familiar enough, and the absence of these same characteristics in those who by nature or art are eunuchs is also well known. These characteristics do not depend upon the production of the normal external secretion—the semen; for it is known that complete virile characteristics are present when the seminal fluid is entirely ineffective by reason of the presence of the condition of aspermatism. Juvenal himself records that men who were possessed of full virility, but without power of fecundation, were sought by the *debauchées* of Imperial Rome, because as the result of their satisfying labours *abortivo non est opus.* Furthermore, Fürbringer, professing to be following Brown-Séquard's method, experimented with seminal fluid, as an orchitic liquid, administered hypodermatically, but reported to the Medical Society of Berlin that no general effects of a tonic or stimulant nature were thereby produced. So far, therefore, as testicular fluid has any action, this action must be due to the presence of the "internal secretion." Again, it has been argued that effects observed after this treatment are the results of "suggestion," an example of the influence of the mind upon the body,—against which view it is urged (1st) that repeated doses of the fluid are required before the effect commences; (2nd) that in experiments made by Variot and others with the genuine extract and with water coloured to resemble it, unknown to the patient, effects were found to follow the use of the organic extract only; and (3rd) Mairet obtained positive results in insane persons who were not open to "suggestion." The enduring (but not immediate) physiological effects of the injection of the orchitic fluid are: stimulation of the mental faculties as by a cerebral tonic; promotion of those functions presided over by the medulla and acceleration of sensory impressions through the spinal cord

[1] *Brit. Med. Journ.*, June 10, 1893.

[2] *La Méthode de Brown-Séquard* (Paris, Baillière, 1893).

[3] *Guide pratique pour la préparation, &c., des liquides organiques, méthode de Brown-Séquard* (Société d'éditions scientifiques, Paris, 1893).

(Grigorescu); increase of muscular force (Copriati); diminished secretion of urine and diminution of vascular tone (Bayroff); stimulation of the function of the digestive tract and frequently augmentation of weight (Bayroff), and increase of the hæmoglobin in the blood. Hence it is easy to deduce that a wide field for application of the treatment is opened out. Omitting Brown-Séquard's classic description of his own case at the Society of Biology in Paris, successful treatment of neurasthenia is recorded by d'Arsonval and others, about one half of the cases so treated being benefited; hysteria has not been found to be relieved; so also in mental alienation, general paralysis and paralysis agitans, the results have been inconclusive. Waterhouse and Pempoukis record benefit in cases of hemiplegia and paraplegia, Deydren in chorea, and Massalongo in insular sclerosis. But it is in locomotor ataxy that the most surprising results have been obtained. Brown-Séquard reported to the Academy of Sciences in April last that, out of 342 cases in which the diagnosis was certain, amelioration to a more or less considerable degree had taken place in 314 cases. Depoux, Gibert, d'Arsonval, Kostourin, Owspenski, Decoud, and Bouffé have also recorded successes. For favourable results the case should be in a comparatively early stage, with at least partial preservation of function; if tolerated, the treatment must be persevered with, the injections administered in series, with periods of intermission over a lengthened space of time. In some cases, however, undue nervous excitement has been caused with increase of the lightning pains, in which case the treatment should of course be discontinued. In tuberculosis, though devoid of specific action, it causes a general improvement in respect of nutrition, night-sweats, cough, and pyrexia, but its use should be avoided in advanced cases. Goizet, Dumont-Pallier, Variot, Lemoine, and others have recorded their experiences. Quite wonderful is the improvement stated to have been observed in 300 cases of the cancerous cachexia, where the lesion was superficial and could therefore be fully observed. The different forms of diabetes mellitus, like anæmia and asthenia, are beneficially influenced, including that special form of diabetes associated with disease of the pancreas; and very many other morbid conditions are benefited by treatment with this substance, such as palpitation, dyspepsia, impotence, general weakness, and so forth. But enough has been adduced to show that in the hands of 1,200 medical men, to whom the College of France has furnished the orchitic extract, results have been obtained which assign a distinct value to this therapeutic measure. Lastly, it is to be remembered in the words of Éloy: "The orchitic extract is always a symptomatic remedy, never a specific remedy."

2. **Thyroid.**—The therapeutic effects of the internal secretion of this gland are so well and so certainly known in this country, applied to the treatment of myxœdema and allied conditions, that it is unnecessary to occupy space here by recording instances; but of the extended range of the influence of this gland-secretion, Dr. Byrom Bramwell's experience in treating psoriasis by thyroid feeding[1] may be noted.

3. **Pancreas.**—The relation of the pancreas to certain forms of diabetes has been discussed previously in this *Journal*,[2] and need not again be stated. It is, however, still uncertain whether we can distinguish clinically in man the cases of diabetes which are wholly or mainly due to the suppression of the internal secretion of the pancreas. They are to be found among the acute rapid examples occurring in the earlier part of life, and it is only in this wasting "*diabète maigre*" that pancreatic treatment is indicated. Many cases have been recorded as having been treated in this country, the pancreas, or a preparation of it, being generally administered by the mouth. On the Continent, Rémond and Rispal, Battistini and Comby have published results more or less variable. The treatment at present is only in a tentative stage, and Brown-Séquard (as before mentioned) has obtained better results in diabetes from the orchitic fluid.

4. **Supra-renal Capsule.**—Experiments by Abelous, Langlois, Albanese, and Charrin having shown that after the capsules have been wholly or mostly removed death takes place, preceded by wasting and great muscular weakness; that this fatal result can be retarded by the introduction into the acapsulated animal of blood obtained from a normal animal, and that this "internal secretion" is allied to curare in its action, some tentative trials have been made in the treatment of Addison's disease by grafting a dog's capsule into the patient by Augagneur, and by injection of the capsular extract by Abelous, Langlois, and Charrin. Huchard has also proposed the same treatment for general muscular weakness. No conclusive results have been hitherto obtained.

5. **Kidney.**—The fact that the condition of anuria is much less rapidly fatal than suppression of the secretions—internal and external—of the kidney when that organ has been ablated, or is structurally diseased, has justified Dieulafoy in mitigating the uræmic state by the hypodermic administration of "nephrine."

6. **Nerve Centres.**—This extract was first employed by Babes, and much more extensively by Constantin Paul for neurasthenia and ataxy. In neurasthenia of the melancholic and hypochondriac type it was found ineffective; in the other forms a large proportion of the cases were benefited. In ataxy Dauriac and Dufournier report twenty out of twenty-four cases more or less ameliorated. Babes and Gibier each report cases of epilepsy which improved under the treatment. Cullère finds that in the insane it produces an improvement in the nutrition

[1] *Brit. Med. Journ.*, Oct. 28, 1893, p. 933.

[2] Sept., 1893, p. 178.

of the body, but no lasting effect upon the mental condition. By other physicians it has been used in aphasia, hemiplegia, and allied conditions with some success; but Brown-Séquard considers that it is distinctly inferior to the orchitic extract.

It would perhaps not be right to omit altogether notice of the use of organic extracts by Hammond,[1] of New York. By a chemical manufacturing company extracts of brain, heart, and other organs are made under his supervision, the effects of which he reports to be remarkable. These effects are denied by Stockwell[2] and others, and, with little credit to those engaged in the wrangle, discussion has been carried on in language and methods happily foreign to this *Journal*.

J. E. Shaw.

### SURGERY.

[1] *N. Y. Med. Journ.*, Jan. 28, Ap. 22, 1893, *etc.*

[2] *Med. News*, Aug. 26, 1893, p. 231, *etc.*

